# Musculoskeletal symptoms among radiologists in Saudi Arabia: a multi-center cross-sectional study

**DOI:** 10.1186/s12891-019-2933-1

**Published:** 2019-11-14

**Authors:** Malak Al Shammari, Ali Hassan, Omran Al Dandan, Mohammed Al Gadeeb, Dalal Bubshait

**Affiliations:** 1 Department of Family and Community Medicine, Imam Abdulrahman Bin Faisal University, Dammam, Saudi Arabia; 2 Department of Radiology, King Fahd Hospital of the University, Imam Abdulrahman Bin Faisal University, Al-Khobar, Saudi Arabia; 3 Department of Orthopedic Surgery, King Fahd Hospital of the University, Imam Abdulrahman Bin Faisal University, Al-Khobar, Saudi Arabia

**Keywords:** Ergonomics, Musculoskeletal symptoms, Radiology, Work-related illness

## Abstract

**Background:**

Musculoskeletal symptoms account for the majority of work-related illnesses resulting in a significant economic burden on society. Computer users are subject to unique repetitive strains that predispose them to musculoskeletal symptoms. In the digitalized field of radiology, radiologists spend long hours interpreting medical images on computers. This study aimed to determine the prevalence of musculoskeletal symptoms among radiologists in Saudi Arabia and their contributing factors.

**Methods:**

An online survey was sent to radiologists in all hospitals (academic, public and private) in the major cities of the Eastern Province of Saudi Arabia covering demographic characteristics, workload (e.g. the time spent at a computer workstation), and workstation environments including the number of monitors as well as the adjustability of the height of the workstation and the viewing distance. This survey of 263 radiologists was conducted in April 2019. It included an evaluation of musculoskeletal symptoms using the Nordic Musculoskeletal Questionnaire. The study outcome was the presence of disabling musculoskeletal symptoms in any body region, which restricted the performance of normal activities in the last 12 months. Results were analyzed descriptively using a Chi-square test and logistic regression analysis to estimate the odd ratio of experiencing disabling musculoskeletal symptoms in the last 12 months.

**Results:**

The survey was completed by 198 participants (111 men and 87 women) with a response rate of 75.3%. Most participants (71.2%) were aged below 40 years. A multivariate logistic regression analysis revealed being a female radiologist (OR = 2.7; 95% CI: 1.2–6.5), aged 30–39 years (OR = 4.1; 95% CI: 1.1–15.3), and predominantly reviewing computed tomography (CT) images (OR = 4.1; 95% CI: 1.4–12.3) or ultrasound scans (OR = 5.9; 95% CI: 1.4–25.3) were associated with higher prevalence of disabling musculoskeletal symptoms, compared to those aged below 30 years and those who reviewed various imaging modalities, respectively.

**Conclusions:**

Musculoskeletal symptoms are common among radiologists with lower back and neck pain being the most frequent complaints. Being a female radiologist, aged 30–39 years, and reviewing CT or ultrasound scans were associated with higher rates of disabling musculoskeletal symptoms.

## Background

Work-related musculoskeletal symptoms refer to muscular, nervous, tendinous, or joint-related discomforts in various parts of the body that are primarily attributable to the work environment, and are worsened or prolonged by working conditions [1]. These musculoskeletal symptoms pose a high economic burden. According to the Global Burden of Disease Study 2016, lower back pain was the leading cause of years of life lost due to disability. Neck pain and other musculoskeletal conditions were the sixth and seventh leading causes, respectively [2]. In the United States, musculoskeletal symptoms represent a significant health problem that accounts for approximately one-third of all work-related illnesses [3]. Between 1996 and 2013, $87.6 billion was spent in the United States for the treatment of neck and lower back pain, making it the third-largest health funding expenditure [4]. Healthcare providers themselves are particularly prone to various musculoskeletal symptoms as a result of from repetitive tasks, performance of high-force manual techniques, and awkward postures [5].

In the last few decades, advances in technology have revolutionized the practice of clinical radiology. These advances include the introduction of picture archiving and communication system (PACS) workstations, speech recognition devices, and electronic medical reports. The transition from hard-copy films to the PACS is beneficial for radiologists and their colleagues, and this translates to better patient care. For example, the use of the PACS has shown to increase both the productivity and diagnostic accuracy, which has resulted in better job satisfaction and more efficient workflow [6, 7]. However, there are some inherent drawbacks to using this technology. The use of PACS have led to decreased direct communication between radiologists and other clinicians and increased the time spent by radiologists seated in their computer workstations [8, 9].

Radiologists are prone to musculoskeletal symptoms due to prolonged computer use [10, 11]. Several cross-sectional studies demonstrated a high prevalence of musculoskeletal symptoms among radiologists. In 2018, Parikh et al. reported that one-quarter of radiologists or radiation oncologists experienced frequent neck pain and approximately one-third experienced lower back pain [12]. A study on breast-imaging radiologists revealed that 60% had experienced musculoskeletal symptoms [13]. Seidel and Krupinski reported that 66% of all participating radiologists experienced neck or lower back pain at least once a week, and those who worked for longer hours reported more pain [9]. With workloads increasing in the radiology sector [14, 15], radiologists may more frequently experience musculoskeletal symptoms. The resulting fatigue and discomfort may lead to errors in the interpretation of medical images [16].

In Saudi Arabia, little is known about the prevalence of musculoskeletal symptoms and the specific risk factors that predispose radiologists to them. To the best of our knowledge, this is the first study to investigate the prevalence of musculoskeletal symptoms among radiologists in Saudi Arabia and to identify the factors contributing to the musculoskeletal discomfort in this group.

## Methods

### Study design

The survey was designed using the QuestionPro survey software (Seattle, WA, USA). As it is easily accessible, saves time, and is cost-effective, an online survey format was selected in our study. The survey was designed to be taken anonymously—without personal identification data requested or stored—and was completed in approximately 7 min.

A cover letter describing the purpose of the study, informing participants of the voluntary nature of their participation, and assuring their anonymity was provided along with the survey questionnaire. Participants were encouraged to contact the research investigator for any queries pertaining to the study, using the provided contact information.

### Study participants

This cross-sectional study was designed to assess work-related musculoskeletal symptoms among clinical radiologists, including residents, specialists (junior staff), and consultants (staff) practicing across all hospitals (academic, public and private) in the major cities of the Eastern Province of Saudi Arabia, which included a total of 12 institutions.

### Recruitment of participants

We sent a personalized message with a link to the online survey to all members (*n* = 110) of a WhatsApp (Facebook, Menlo Park, CA, USA) group of radiology residents practicing in the Eastern Province. The link to the survey was also sent to radiology specialists and consultants whose contact information was available to the investigators. A reminder message was sent three days later. The survey began on April 28, 2019, and was open to respondents for 14 days.

Each invited radiologist received a unique link for the online survey so that the survey could not be filled more than once from the same link. This ensured that the survey would not be compromised by duplicate responses or responses from individuals not included in the target population. Anonymity of the respondents’ identity was maintained using the QuestionPro respondent anonymity assurance feature.

Additionally, a paper-based survey questionnaire was distributed to the radiology departments of hospitals in the surveyed region to reach radiologists whose contact information was unavailable with the investigators. Investigators visited those departments a week later to collect the completed surveys. Overall, the survey was distributed to a total of 263 radiologists using both modes (online and paper).

### Content of the questionnaire

The survey was comprised of 25 multiple-choice questions covering the following areas: (1) background demographic information, (2) work-related data, (3) workstation evaluation, and (4) identification of work-related musculoskeletal symptoms. There were questions asking about the physical activity and the methods used by radiologists to generate radiology reports; the results of these questions are outside the scope of the current study and will be reported separately. We conducted a pilot study with a group of 30 radiologists to assess the clarity of the questions and the time needed to complete the survey. After the pilot study, no major changes were made to the questions.

#### Exposure variables

The proposed risk factors were determined based on the literature focusing on individual demographics and work-related information. Background demographic information included data pertaining to age group (< 30 years, 30–39 years, 40–49 years, 50–59 years, and ≥ 60 years), sex, handedness, years of practice (< 1 year, 1–5 years, 5–9 years, and ≥ 10 years), the current institution of practice, and the type of practice (part-time, full-time, or both). Work-related characteristics included the time spent at a computer workstation reviewing medical images (< 4 h, 4–7 h, 7–9 h, and >  9 h), the amount and type of imaging studies typically reviewed (plain radiography, ultrasound, computed tomography (CT), magnetic resonance imaging (MRI), fluoroscopy, and nuclear medicine), as well as the duration (< 5 min, 5–10 min, 11–15 min, > 15 min) and frequency of breaks taken (once, twice, every 2 h, and at least every hour). Information about the average percentage of time spent on each imaging modality was grouped based on the time distribution (0%, 1–25%, 26–50%, 51–75%, and 76–100%). For example, if a participant spent more than 75% of his or her working time on a particular imaging modality, this modality was considered as the predominantly reviewed modality for the participant. A Likert-like scale (always, often, sometimes, rarely, and never) was used to record data about the performance of the workstation stretching exercises by participants. In addition, the workstation was evaluated in terms of the number of monitors and the adjustability of the height of the workstation and the viewing distance (adjustable vs. nonadjustable).

#### Outcome variables

The standard Nordic Musculoskeletal Questionnaire, a valid and reliable screening and surveillance tool [17], was used to determine which body regions were affected by musculoskeletal symptoms resulting from working as a radiologist. It included the following questions about nine body regions (neck, shoulder, elbow, wrist/hand, upper back, lower back, hip/thigh/buttock, knee, and ankle):

1. Have you had trouble (ache, pain, or discomfort) in the last 12 months?
2. Have you had trouble in the last 7 days?
3. Have you been prevented from carrying out normal activities (e.g., job, housework, or hobbies) due to this trouble in the last 12 months?

In this study, the outcome was the presence of musculoskeletal symptoms in any of the nine body regions, which restricted the performance of normal activities in the last 12 months. The responses of the outcome variables were dichotomized: responses of “left”, “right” or “bilateral” in any body region were coded as a “yes”, whereas a respondent who indicated “no” for all body regions was coded as a “no”.

### Statistical analysis

The obtained data were compiled using the QuestionPro platform and analyzed using IBM SPSS for Windows, version 25 (IBM Corp., Armonk, NY, USA). Two questionnaires were excluded from the analysis because of missing data. All variables used in the study were categorial. Descriptive statistics, such as percentages and frequency distribution of different characteristics, were used as appropriate. In questions based on a Likert-type scale, the responses were combined into two responses. The Chi-square test was used to confirm the bivariate relationship between the explanatory and outcome variables. A multivariable logistic regression analysis was performed to identify independent factors associated with musculoskeletal symptoms. Candidate variables were selected based on biologic plausibility, risk factors that have been identified in the literature, and bivariate analysis results. Manual backward stepwise regression was used to eliminate covariates whose inclusion did not significantly improve the Akaike Information Criterion for the model. In addition, the regression model was tested for multicollinearity using the variance inflation factor statistic. The result of the modeling process was a single multivariate model that encompassed all potential factors relating to the outcome variable. The covariates included age, sex, current institution of practice, and time spent at a computer workstation reviewing medical images. The unadjusted and adjusted odds ratios (OR) with their 95% confidence interval (95% CI) were reported in comparison to the designated referent. When estimating the OR for participants who predominantly review and interpret a particular imaging modality, those who spent their time on different modalities were used as the reference group. The statements of statistical significance were based on a significance level of α = 0.05.

## Results

### Characteristics of the participants

A total of 198 participants completed the survey with a response rate of 75.3% (198/263). Overall, there were 81 (40.9%) residents, 54 (27.3%) specialists, and 63 (31.8%) consultants. Of the 198 participants who responded, 111 (56.1%) were men and 87 (43.9%) were women. Most of the participants belonged to the 30–39 years and <  30-year-age groups, with 80 (40.4%) and 61 (30.8%) participants, respectively. In addition, 72 (36.4%) participants had 1–5 years of practice, while 67 (33.9%) had over 10 years of practice (Table 1).

**Table 1:** Characteristics of the participants

Characteristics		Number of Participants (%)	
Age (years)	< 30	61	(30.8)
	30–39	80	(40.4)
	40–49	35	(17.7)
	50–59	15	(7.6)
	≥ 60	7	(3.5)
Gender	Male	111	(56.1)
	Female	87	(43.9)
Handedness	Right-handed	187	(94.4)
	Left-handed	11	(5.6)
Professional Rank	Resident	81	(40.9)
	Specialist	54	(27.3)
	Consultant	63	(31.8)
Years in Practice	Less than 1	20	(10.1)
	1 to 5	72	(36.4)
	6 to 10	37	(19.7)
	More than 10	67	(33.8)
Institution	Academic	33	(16.7)
	Public	124	(62.6)
	Private	41	(20.7)

### Work environment of the participants

Table 2 summarizes the workload and work environment of the participants. Most participants (114, or 57.6%) spend 7–9 h daily at computer workstations reviewing images, and only 50 (25.3%) take a break once daily. Overall, 20.7% of the men take breaks of more than 15 min, while 10.3% of the women take a break of more than 15 min (*P* = 0.046).

**Table 2:** Work environment of the participants

Characteristics		Male (%)		Female (%)		Total
Time Spent at Computer Workstation (hours/day)	< 4	5	(62.5)	3	(37.5)	8
	4–6	39	(59.1)	27	(40.9)	66
	7–9	63	(55.3)	51	(44.7)	114
	> 9	4	(40.0)	6	(60.0)	10
Frequency of Breaks	Once/day	24	(48.0)	26	(52.0)	50
	Twice/day	33	(54.1)	28	(45.9)	61
	Every 2 h	38	(59.4)	26	(40.6)	64
	At least hourly	16	(69.6)	7	(30.4)	23
Duration of Breaks (min)	< 5	7	(33.3)	14	(66.7)	21
	5–10	48	(53.9)	41	(46.1)	89
	11–15	33	(58.9)	23	(41.1)	56
	> 15	23	(71.9)	9	(28.1)	32
Workstation Stretching Exercises	Yes	71	(55.5)	57	(44.5)	128
	No	40	(57.1)	30	(42.9)	70
Number of Monitors	One	5	(45.5)	6	(54.5)	11
	Two	40	(59.7)	27	(40.3)	67
	Three or more	63	(57.3)	47	(42.7)	110
	Varies	3	(30.0)	7	(70.0)	10
Adjustable Height of Workstation?	Yes	74	(58.3)	53	(41.7)	127
	No	37	(52.1)	34	(47.9)	71
Easily Adjustable Viewing Distance?	Yes	76	(58.9)	53	(41.1)	129
	No	35	(50.7)	34	(49.3)	69

Overall, 110 (55.6%) participants had 3 or more monitors at their workstation. A total of 129 (65.2%) participants indicated that they can easily adjust their viewing distance while 71 (35.9%) participants reported that the height of their workstation cannot be adjusted. However, of those having workstations with adjustable height, 24.4% reported they had never made their personal adjustments.

There was no statistically significant difference between men and women with regards to the percentage of time spent on each type of imaging study, with the exception of ultrasound scans where women spent over 50% of their time in a week as compared to men (*P* = 0.048).

### Musculoskeletal symptoms among the participants

Among all respondents, 140 (70.7%) participants had musculoskeletal symptoms occurring in at least one body region during the 7 days preceding the study. Overall, 176 (88.9%) participants reported having symptoms in the last 12 months preceding the study, with 58 (29.3%) participants being prevented from performing normal activities due to these symptoms (Table 3).

**Table 3:** Musculoskeletal symptoms among participants

Body Part		Symptoms in the Past 7 days		Symptoms in the Past 12 months		Disabling Symptoms in the Past 12 months	
		N	(%)	N	(%)	N	(%)
Any Part		140	(70.7)	176	(88.9)	58	(29.3)
Neck		81	(40.9)	124	(62.6)	30	(15.2)
Shoulders	Any	64	(32.3)	109	(55.1)	29	(14.6)
	Right	18	(9.1)	42	(21.2)	8	(4.0)
	Left	13	(6.6)	17	(8.6)	7	(3.5)
	Bilateral	33	(16.7)	50	(25.3)	14	(7.1)
Elbows	Any	18	(9.1)	38	(19.2)	8	(4.0)
	Right	13	(6.6)	19	(9.6)	7	(3.5)
	Left	4	(2.0)	8	(4.0)	0	(0.0)
	Bilateral	1	(0.5)	11	(5.6)	1	(0.5)
Wrists/Hands	Any	49	(24.7)	109	(55.1)	16	(8.1)
	Right	35	(17.7)	79	(39.9)	11	(5.6)
	Left	6	(3.0)	9	(4.5)	4	(2.0)
	Bilateral	8	(4.0)	21	(10.6)	1	(0.5)
Upper Back		56	(28.3)	97	(49.0)	19	(9.6)
Lower Back		84	(42.4)	137	(69.2)	29	(14.6)
Hips/Thighs/Buttocks		33	(13.6)	68	(34.3)	14	(7.1)
Knees		27	(13.6)	50	(25.3)	14	(7.1)
Ankles		11	(5.6)	19	(9.6)	4	(2.0)

*N* number of participants

The musculoskeletal symptoms varied according to affected body area. Within the 7 days preceding the study, a substantial proportion of the participants reported lower back (42.4%), neck (40.9%), or shoulder (32.3%) symptoms. Almost one-quarter (24.7%) of the participants had wrist/hand pain during the same period. Similarly, these regions were the most frequently affected in the 12 months preceding the study. However, the musculoskeletal symptoms that prevented the participants from performing normal activities in the 12 months preceding the study showed a slightly different pattern, with neck pain being the most common symptom (15.2%).

### Factors related to musculoskeletal symptoms

Musculoskeletal symptoms episodes that restricted the participants’ normal activities in the 12 months preceding the study were associated with several demographic characteristics and work conditions (Table 4). Female sex was associated with a higher prevalence of such disabling symptoms (X^2^ = 4.20, *P* = 0.04), as more women than men reported disabling symptoms in at least one body region [32 (36.8%) females vs. 26 (23.4%) males].

**Table 4:** Univariate and multivariate logistic regression analysis showing the odd ratios for independent factors associated with disabling musculoskeletal symptoms

Variables		Disabling Symptoms in the Past 12 months? (*N* = 58)	Univariate			Multivariate	
			X^2^	*P*-value ^a^	Unadjusted OR (CI)	Adjusted OR (CI) ^b^	Adjusted P-value ^a^
Age (years)	< 30	12	16.38	**0.003**	1	1	
	30–39	31			2.6 (1.2–5.6)	4.1 (1.1–15.3)	**0.04**
	40–49	4			0.5 (0.2–1.8)	0.5 (0.1–3.7)	0.49
	50–59	7			3.6 (1.1–11.8)	4.7 (0.6–38.4)	0.15
	≥ 60	4			5.4 (1.1–27.6)	6.9 (0.5–90.6)	0.14
Sex	Male	26	4.20	**0.04**	1	1	
	Female	32			1.9 (1.1–3.5)	2.7 (1.2–6.5)	**0.02**
Professional Rank	Consultant	17	1.25	0.54	1	1	
	Specialist	19			1.5 (0.7–3.2)	0.9 (0.3–3.3)	0.89
	Resident	22			1.1 (0.5–2.1)	3.5 (0.3–38.4)	0.29
Years of Practice	Less than 1	4	1.05	0.79	1	1	
	1 to 5	22			1.8 (0.5–5.9)	3.1 (0.7–14.5)	0.15
	6 to 10	11			1.6 (0.4–5.8)	2.7 (0.5–29.4)	0.40
	More than 10	21			1.8 (0.5–6.1)	10.5 (0.8–142.1)	0.08
Time Spent at Computer Workstation (hours/day)	< 4	1	10.69	**0.014**	1	1	
	4–6	11			1.4 (0.2–12.5)	0.08 (0.03–1.7)	0.11
	7–9	41			3.9 (0.5–33.1)	0.2 (0.02–1.1)	0.06
	> 9	5			7.0 (0.6–79.9)	0.5 (0.9–3.0)	0.45
Frequency of Breaks	At least every hour	4	3.51	0.32	1	1	
	Every 2 h	18			1.9 (0.6–6.2)	1.6 (0.4–7.3)	0.54
	Twice a day	17			1.8 (0.6–6.2)	1.2 (0.4–3.7)	0.80
	Once a day	19			2.9 (0.9–9.9)	1.9 (0.6–6.4)	0.31
Duration of Breaks (min)	< 5	9	3.01	0.39	1.9 (0.6–6.1)	1.6 (0.5–17.3)	0.44
	5–10	22			0.8 (0.3–2.1)	1.2 (0.5–3.8)	0.90
	11–15	18			1.2 (0.5–3.1)	1.3 (0.3–9.4)	0.41
	> 15	9			1	1	
Workstation Stretching Exercises	No	33	2.16	0.14	1	1	
	Yes	25			0.6 (0.3–1.2)	0.7 (0.4–1.4)	0.28
Adjustable Height of Workstation?	Yes	15	3.56	0.06	1	1	
	No	43			1.9 (1.0–3.8)	1.7 (1.0–3.9)	0.13
Easily Adjustable Viewing Distance?	Yes	36	0.34	0.56	1	1	
	No	22			1.2 (0.6–2.3)	2.0 (0.8–4.9)	0.12
Imaging Modality Predominantly Reported	CT	10	15.92	**0.007**	2.9 (1.1–7.1)	4.1 (1.4–12.3)	**0.01**
	MRI	2			3.7 (0.3–60.9)	4.9 (0.2–120.0)	0.33
	US	8			5.6 (1.8–16.8)	5.9 (1.4–25.3)	**0.02**
	Nuclear Imaging	3			3.7 (0.7–19.3)	9.0 (0.9–89.4)	0.06
	Fluoroscopy	3			[Cannot Be Computed]		0.99
	Varies	30			1	1	

*CT* computed tomography; *MRI* magnetic resonance imaging; *US* ultrasound

^a^P-value in bold when significant

^b^ORs are adjusted for age, sex, institution of practice, and time spent at computer workstation

Participant age was also an important factor (X^2^ = 16.38, *P* = 0.003). Overall, 47.8% of participants ≥50 years old reported at least one disabling musculoskeletal symptom in the year preceding the survey, compared with 19.7% of those < 30 years. Specifically, participants aged 50–59 years and ≥ 60 years reported disabling symptoms 3.6- and 5.4-times more frequently, respectively, than participants < 30 years (*P* < 0.05).

The average daily hours spent by radiologists at computer workstations reviewing medical images was significantly associated with the presence of disabling musculoskeletal symptoms (X^2^ = 10.69, *P* = 0.014). Out of the total respondents who spent 7–9 h daily at computer workstations, 41 (36%) participants reported an episode of disabling symptoms. Half of the participants who spent over 9 h daily at computer workstations had an episode of disabling symptoms. The practice of spending 7 h or more daily at computer workstations was significantly associated with an increase in disabling symptoms among the participants (*P* < 0.05).

As only 13 (6.57%) participants reported that they routinely performed stretching exercises at the workstation, we could not evaluate the extent of the impact of these exercises on the prevalence of musculoskeletal symptoms among all the participants.

The imaging modality predominantly reviewed by radiologists was associated with the presence of disabling musculoskeletal symptoms (X^2^ = 15.95, *P* = 0.007). Prevalence of musculoskeletal symptoms was higher among participants who spent most of their time reviewing CT (43.5%), MRI (50.0%), ultrasound (44.4%), nuclear medicine (50.0%), and fluoroscopy (50.0%) compared to those who reviewed various imaging modalities (21.3%).

There were no statistically significant associations between the occurrence of musculoskeletal symptoms and the place of work, number of years in practice, professional rank, frequency and duration of breaks, and adjustability of the viewing distance or the workstation height.

### Multivariate analysis for the factors associated with musculoskeletal symptoms

A multivariate logistic regression analysis revealed that women were at 2.7 times higher risk of having disabling musculoskeletal symptoms resulting from working as a radiologist (95% CI: 1.2–6.5). The analysis revealed that age group (30–39 years) parameter behaved as an independent factor associated with musculoskeletal symptoms (OR = 4.1; 95% CI: 1.1–15.3). In addition, radiologists who predominantly reviewed CT images (OR = 4.1; 95% CI: 1.4–12.3) and ultrasound scans (OR = 5.9; 95% CI: 1.4–25.3) were at a higher tendency of exhibiting musculoskeletal symptoms in the 12 months preceding the survey (Table 4).

## Discussion

Our study reported a high prevalence of musculoskeletal symptoms among radiologists, with up to 70.7% of the participating radiologists experiencing musculoskeletal symptoms in the week preceding the survey. This finding is consistent with those of previously published studies reporting a high prevalence of musculoskeletal symptoms among radiologists [9, 13, 18]. The significant contributing factors to musculoskeletal symptoms included age, sex, hours spent at a computer workstation reviewing medical images, and the type of medical images predominantly reviewed.

Lower back pain was the most frequently reported musculoskeletal symptom among participating radiologists in the 12 months preceding the study. This prevalence was significantly higher than the global prevalence of lower back pain based on a systematic review, including 165 population studies, which reported a 1-year prevalence of 38% [19]. The Global Burden of Disease Study 2016 identified both obesity and occupational environment are associated with an increased prevalence of lower back pain [2]. Upper extremity musculoskeletal conditions, including carpal tunnel syndrome and tenosynovitis, have been observed among radiologists [11, 20]. In our study, almost half of the participants reported some pain or discomfort involving the hands/wrists in the year preceding the study, which might have been related to the use of computers or handheld devices.

Neck pain was the second most prevalent musculoskeletal symptom among our participants in the 12 months preceding the survey, and 15.2% experienced neck pain that restricted their activities of daily living. A systematic search and critical literature review of studies published between 1980 and 2006 reported the 12-month prevalence of neck pain to typically range from 30 to 50% [21]; it also revealed a higher prevalence among healthcare providers. Furthermore, a study investigating the extent and severity of musculoskeletal symptoms among office workers in a large telecommunication company revealed that the prevalence of neck pain or discomfort reached 77.5% [22]. This finding may reflect the influence of the occupational environment on musculoskeletal symptoms, which was also reported in a similar study on computer users [23].

Female radiologists were more likely to report musculoskeletal symptoms in our study. One of the reasons for this was that radiologists who spent most of their time performing ultrasound scans were females; in our study, radiologists performing ultrasound scans were at a higher risk than radiologists reviewing other imaging modalities. Another study, which also reported that female radiologists were likely to report musculoskeletal symptoms, reported that the design of workstations may favor the male body habitus [9]. The finding of higher prevalence of musculoskeletal symptoms among women is consistent with that of different studies that investigated musculoskeletal symptoms [19, 23, 24]. Various factors have been suggested in an attempt to explain sex differences in the prevalence of musculoskeletal symptoms, including physiological differences in terms of muscle and bone mass and psychological differences such as the likelihood of reporting somatic symptoms [19].

We found that the prevalence of musculoskeletal symptoms increases with aging. This is expected, considering the impact of age-related degenerative changes. However, in our study, the prevalence of musculoskeletal symptoms severe enough to restrict performance of normal activities was reported to be 19.7% among radiologists aged less than 30 years. This rate is high considering that 30.8% of our study participants were in that age group. Indeed, being in age group of 30–39 years was independent factor associated with experiencing musculoskeletal symptoms. It is likely that these young radiologists who had disabling musculoskeletal symptoms, despite the short duration of practice, will be prone to recurrent symptoms in the future, unless effective interventions are timely initiated.

Long hours spent sitting at a computer workstation can have deleterious effects on the health of radiologists and may affect patient care. Studies have reported that the diagnostic accuracy of radiologists decreases after they spend 8 h at the workstation [25–27]. Higher prevalence of musculoskeletal symptoms was reported among radiologists who spend a longer time at the radiology workstations [9]; similar findings were also noted in our study. The majority of the participants in the present study spent 7–9 h daily in front of a computer reviewing medical images, which is consistent with the findings of a previous study on radiologists [18]. A meta-analysis of 6 studies involving a total of almost 600,000 adults reported that higher durations of daily total sitting time are associated with a higher risk of all-cause mortality [28]. Subsequent studies have also emphasized this association [29]. To prevent such adverse effects, it is advised that radiologists should perform stretching exercises at their workstations at least hourly [30]. In our study, however, the majority of the radiologists did not routinely perform stretching exercises at their workstations and did not take timely breaks during the day.

Considering the nature of imaging modalities that radiologists predominantly interpreted and reported during their daily practice, we found that those who spent most of their time reviewing a particular imaging modality had higher prevalence of disabling musculoskeletal symptoms compared to those who did not. First, we postulated that radiologists who predominantly reviewed a single imaging modality were most likely to be consultant radiologists and older in age compared to those who review different imaging modalities. However, a multivariate logistic regression analysis showed that those who spent most of their time reviewing CT images or ultrasound scans had the highest rates of musculoskeletal symptoms.

The increased workload in the field of radiology and the advent of novel technologies have resulted in a significant increase in the number of CT images generated per radiological examination [14, 31]. Therefore, it is possible that radiologists reviewing CT scans are required to read and analyze an increasing number of digital images at computer workstations, thereby succumbing to occupational-related musculoskeletal symptoms.

The association between sonography and the risk of developing musculoskeletal problems is well-established [32]. As high as 95% of all sonographers experience work-related musculoskeletal symptoms, of whom 90% experience pain for more than half of their careers [33, 34]. The average duration of musculoskeletal symptoms is 52 months [35]. Moreover, approximately 20% of sonographers end their careers as a result of work-related symptoms [36]. In our study, multivariate analysis showed that radiologists who predominantly perform ultrasound scanning had 5.9 times higher tendency for musculoskeletal symptoms. Notably, although sonographers were available to perform ultrasound scans at all institutions we studied, radiologists also performed scans as part of their training and practice. Boiselle et al. demonstrated that 55% of radiologists spent more than 2 h per day in awkward positions [18].

Several factors predispose sonographers to musculoskeletal pain, which include the need for sonographers to perform fine, repetitive, and sometimes forceful movements that necessitate maintaining awkward postures [33]. The movements are often more difficult when examining obese patients, which usually requires examiners to push a transducer into patients to achieve the desired image; this increases examination times and the time required to maintain awkward positions [37]. The optimization of ultrasound images should not come at the expense of practitioners’ health. Thus, sonographers are recommended to spend a few minutes prior to each exam to optimize equipment and the patient’s positions so as to minimize muscle strain [38].

Ergonomics, the science concerned with the understanding of an individual’s interaction with the workplace and environment, aims to reduce stress, eliminate injuries resulting from repeated tasks and incorrect posture, and maximize productivity [39]. Some studies have addressed ergonomic strategies to reduce musculoskeletal symptoms among radiologists. Rodrigues et al. demonstrated a high prevalence of musculoskeletal symptoms in radiology workstations that show poor compliance with ergonomic best practices [40]. Boiselle et al. showed that although musculoskeletal symptoms are highly prevalent among radiologists, ergonomic interventions can effectively reduce these symptoms [18]. A previous study demonstrated that ergonomic devices and ergonomic training may help prevent musculoskeletal symptoms among radiologists working in breast imaging [13]. Unexpectedly, our study showed no significant association between the adjustability of the height of the workstation and the viewing distance with musculoskeletal symptoms. However, multiple reasons can explain this observation. First, the radiology workstations in the hospitals included in this study were not uniform; therefore, the ergonomic features may vary within the same workplace. Second, a major proportion of the study participants included radiology residents, who underwent frequent rotations in different hospitals, with exposure to variable workstations as part of their training programs, which complicated the investigation of the ergonomic impact of workstations. Third, it should be emphasized that the ergonomic interventions might be ineffective if the participants are not imparted any education and training on ergonomics. For example, around 25% of radiologists in our study reported never making personal adjustments of workstation height despite an adjustable workstation. One study reported that radiologists with good knowledge of ergonomics experienced significantly less lower back pain compared to those with poor knowledge [40]. Furthermore, the same study demonstrated that even where certain facilities were available, less than a third of radiologists made personal adjustments prior to a reporting session.

This study is the first to investigate musculoskeletal symptoms among radiologists in Saudi Arabia. Moreover, our study included all hospitals in major cities of the Eastern Province, which is the largest governorate in Saudi Arabia. However, despite these strengths, the present study has certain limitations. The musculoskeletal symptoms were self-reported. Although self-reporting can be rapid and convenient, it may also introduce bias such as those who experienced musculoskeletal symptoms were more likely to respond than those who did not. In addition, the present study was a cross-sectional survey; therefore, causality could not be assessed directly. Furthermore, complaints of musculoskeletal symptoms are common in the general population; all reported symptoms cannot be attributed to work environments. However, we had emphasized on the cover page of the survey that only work-related symptoms should be reported by the participants. Further, a comparison group would have enabled a more accurate evaluation of work-related musculoskeletal symptoms among radiologists. Lastly, our study did not consider the interaction between physical, psychological, and social factors.

The high prevalence rate among our study participants warrants the consideration for implementing preventive interventions. Such measures need to be developed with the aims of increasing the awareness of this issue among practicing radiologists, followed by educating the radiologists regarding the adverse effects of prolonged computer use, and imparting training to ensure proper ergonomic practices. Further studies incorporating proper assessments and physical examinations with objective evaluation of workstation ergonomics are recommended.

## Conclusions

Musculoskeletal symptoms are common among radiologists with lower back and neck pain being the most frequent complaints. Being a female radiologist, aged 30–39 years, and reviewing CT and ultrasound scans were associated with higher rates of musculoskeletal symptoms. The results of this study can be used to develop intervention strategies to reduce the incidence of musculoskeletal symptoms among clinical radiologists.

## Data Availability

The datasets used and/or analyzed during the current study are available from the corresponding author on reasonable request.

## Abbreviations

CI: Confidence Interval
CT: Computed Tomography
OR: Odd Ratio
PACS: Picture archiving and communication systems
